# Analysing the impact of the COVID-19 outbreak on everyday travel behaviour in Germany and potential implications for future travel patterns

**DOI:** 10.1186/s12544-021-00486-2

**Published:** 2021-05-01

**Authors:** Viktoriya Kolarova, Christine Eisenmann, Claudia Nobis, Christian Winkler, Barbara Lenz

**Affiliations:** German Aerospace Center, Institute of Transport Research, Rudower Chaussee 7, 12489 Berlin, Germany

**Keywords:** COVID-19, Travel behaviour, Coronavirus impacts, Home-office, Travel patterns change, Representative survey

## Abstract

**Introduction:**

The global Coronavirus (COVID-19) pandemic is having a great impact on all areas of the everyday life, including travel behaviour. Various measures that focus on restricting social contacts have been implemented in order to reduce the spread of the virus. Understanding how daily activities and travel behaviour change during such global crisis and the reasons behind is crucial for developing suitable strategies for similar future events and analysing potential mid- and long-term impacts.

**Methods:**

In order to provide empirical insights into changes in travel behaviour during the first Coronavirus-related lockdown in 2020 for Germany, an online survey with a relative representative sample for the German population was conducted a week after the start of the nationwide contact ban. The data was analysed performing descriptive and inferential statistical analyses.

**Results and Discussion:**

The results suggest in general an increase in car use and decrease in public transport use as well as more negative perception of public transport as a transport alternative during the pandemic. Regarding activity-related travel patterns, the findings show firstly, that the majority of people go less frequent shopping; simultaneously, an increase in online shopping can be seen and characteristics of this group were analysed. Secondly, half of the adult population still left their home for leisure or to run errands; young adults were more active than all other age groups. Thirdly, the majority of the working population still went to work; one out of four people worked in home-office. Lastly, potential implications for travel behaviour and activity patterns as well as policy measures are discussed.

## Introduction

The global Coronavirus (COVID-19) pandemic is having a great impact on all areas of the everyday life of people worldwide including travel behaviour. Countries have been implemented various measures that focus on restricting social contacts and reducing in this way the spread of the virus.

In 2020, in most of the federal states in Germany kindergarten, schools and universities have started closing on March 16th. The German Foreign Office issued a worldwide travel alert and warns against unnecessary travel abroad on March 17th; border controls and entry ban were introduced at the borders to France, Austria, Luxembourg, and Switzerland. Few days later, on March 22nd, the German Government announced a nationwide Corona-related contact ban with wide implications for the daily life of the population, including closed restaurants, cafés and also hairdresser, only two persons from different households being allowed to meet in public, and for many of the German working population, when possible, work from home. This first lockdown was extended on April 1st to April 19th and was accepted, i.e. implemented, by all federal states. As the German Government started to relax the Coronavirus-relared restrictions, Saxony was the first federal state in Germany which made face covering mandatory in shops and when using public transportation on April 20th. The other states followed its example.

Mobile phone data analyses show for instance a decrease in trip rates by 39% in the strictest lockdown period between end of March and beginning of April [20]. Further studies suggest that in the same period around one quarter (25%) of the working population in Germany worked in home-office, up to 10% worked short-time [17] and about 69% of the population over 18 had as a results of the restrictions no physical contact at all to friends, relatives and/ or colleagues - compared to 17% at the beginning of March [4]. Moreover, the number of people who purchase groceries at least occasionally online doubled - from 16% before to 30% after the Corona outbreak [3]. These numbers give first insights into changes of daily activity- and travel-related behaviour due to the Corona crisis. However, because of the novelty of the situation, still little is known about how different user groups adapted their daily mobility and travel behaviour, the reasons behind, beyond the government restriction, and the potential implications for travel patterns after the Corona crisis. Besides analysing potential mid- and long-term impacts, understanding how daily activities and travel behaviour change during such global crisis and the reasons behind is crucial for developing suitable strategies for similar future events.

The effects of the Coronavirus-spread on activity and travel behaviour patterns have been already addressed in early studies in different countries around the world (e.g. [1, 2, 8, 10, 11, 14, 16]). Most of the studies looked at travel pattern changes as well as changes in working and shopping behaviours (including working in a home-office and e-commerce, i.e. online shopping). Various methods have been applied, e.g. online surveys (e.g. [8, 10]) and objective data measures via GPS Logger and Travel Diary App (e.g. [2]). Further research works focus on the implications of the global pandemic for the relation between transport and wellbeing in the context of the pandemic as well as for the future agenda for policy and practice. For instance, Musselwhite et al. [18] discussed the link between travel behaviour, especially hypermobility or active travel, and public health that became more present in the event of Coronavirus outbreak and derived policy implications based on this discussion. Cartenì et al. [7] analysed the role of transport accessibility within the spread of the Coronavirus and proposed tailored policy strategies for managing the spread of the virus depending on the accessibility level of the area. A study conducted by Budd and Ison [6] proposed the concept of *Responsible Transport* which is a transport policy approach that considers besides environmental aspects also public health and wellbeing issues. All in one, the mentioned studies stress the importance of looking deeper into the effect of the pandemic on travel behaviour in order to develop more robust and sustainable transport policy and practice strategies and measures.

While there are various studies for other countries, for Germany, there is to our best knowledge a lack of published results from representative studies which focus explicitly on the impact of the first Coronavirus-related lockdown on the travel behaviour.

For these reasons, this study focusses on the changes in travel behaviour patterns during the Coronavirus pandemic and discusses potential mid-and long-term impact on everyday travel patterns for Germany. The following research questions were addressed: (i) how did activity patterns and travel behaviour changes during the first strictest Coronavirus-related lockdown period (with view on shopping, leisure, and working and commuting) compared to the time before the Coronavirus outbreak/spread?; (ii) what are the (potential) reasons behind and which segments are most prone for certain changes?; (iii) which initial conclusions can we derive for potential mid- and long-term impacts of these changes?.

## Methods

In order to provide empirical insights into changes in travel behaviour during the Coronavirus pandemic for Germany, an online survey was conducted from April 6th to April 10th 2020 (during the strictest period of Coronavirus-related lockdown in 2020; a week after the announcement of the nationwide contact ban).^1^ The sample consists of 1.000 participants and is chosen to be representative for the German population between 18 and 82 years in terms of having a sufficient share of people in certain age as well as of certain gender, education level and residential location to represent these segments in the German population. The participants were recruited using the professional panel provider KANTAR GmbH.^2^ The sample was weighted in order to ensure that representative conclusions can be derived. Weighted criteria were the following: gender, age, educational level, spatial type (defined using the statistical spatial typology RegioStaR; [5]), and federal state place of residence. Table 1 gives an overview of selected characteristics of the sample (socio-demographic and travel behaviour-related characteristics).

**Table 1:** Descriptive statistics of selected characteristics of the sample

Variable	Characteristics	Share of respondents [***N*** = 1.000]
Gender	Male	51%
	Female	49%
Age	18–24	12%
	25–34	14%
	35–44	15%
	45–54	16%
	55–64	18%
	65 or above	25%
Educational level	No degree (yet)	2%
	Secondary general school	27%
	Secondary school	31%
	Academic secondary school	17%
	University	17%
	Other education	7%
Employment status	Full-time employed (35 h/ week)	36%
	Part-time employed (18 to 35 h/ week)	11%
	Marginally employed (11 to 18 h/ week)	4%
	Student / trainee	10%
	Retired	30%
	Other employment, unemployed or temporary exemption from work (e.g. parental leave)	9%
Driving license	Percentage of possession	82%
Car in household	Percentage of possession (at least 1 car)	79%
Place of residence - regional type	Urban region	63%
	Rural region	37%
Place of residence - state	Baden-Württemberg	13%
	Bavaria (Bayern)	18%
	Berlin	4%
	Brandenburg	3%
	Bremen	0.4%
	Hamburg	1%
	Hesse (Hessen)	7%
	Lower Saxony (Niedersachsen)	9%
	Mecklenburg-Vorpommern	2%
	North Rhine-Westphalia (Nordrhein-Westfalen)	21%
	Rhineland-Palatinate (Reinland-Pfalz)	5%
	Saarland	1%
	Saxony (Sachsen)	5%
	Saxony-Anhalt (Sachsen-Anhalt)	3%
	Schleswig-Holstein	3%
	Thuringia (Thüringen)	3%

The study focused on general and activity-related (working and commuting, shopping, leisure) travel behaviour before the Coronavirus outbreak/ spread (usual daily behaviour) and during the strictest Coronavirus-related lockdown (i.e. in the time period around the study time point) as well as on attitudes, perceptions and individual coping strategies related to the current situation. The questionnaire included mainly closed questions and few open questions which focused on exploring individual opinions or reasons behind certain behaviour. The attitudinal questions were measured on a five-point Likert scale [15] ranging from *1 = “totally agree”* to *5 = “totally disagree”.* For the analyses of changes in mode choices during the Coronavirus-related lockdown compared to the time before the pandemic, the respondents were divided into “modal groups” depending on the mode(s) of transportation that they used on a certain trip following a methodology proposed by Nobis [19]. A criterion for the classification of respondent to a certain group was whether he/ she uses car, public transport, bicycle or a combination of different modes of transportation. Overall three monomodal groups (car, public transport, and bicycle users) and two multimodal groups (users of various modes of transportation including a car and users of various modes of transportation without a car) were defined.

The data was analysed performing descriptive and inferential statistical analyses looking into changes in activities, mode choices and subjective evaluation of the lockdown situation with regard to mobility and daily activity patterns. For selected open questions (e.g. questions on benefits and disadvantages of home-office), qualitative analyses were performed by categorizing the mentioned aspects in topics. For the analyses of shopping patterns, a binary logistic regression analysis was performed in order to examine the effect of various individual characteristics on the probability to purchase products online instead of in stores during the Coronavirus pandemic. The binary logistic regression was chosen as the dependent variable (with the possible expression “purchasing online instead of in stores” vs. “purchasing in stores”) was assumed to be a categorical variable, i.e. a type of variable which divide the sample in two disjunctive categories ([12], p. 104). An ordinal regression could be also a suitable method if we assume that the dependent variable represents a rang or ordered categories, e.g. ranking people who shop in stores instead of online vs. those who do not. This approach, however, would not allow us to define the characteristics of a certain group (e.g. “online shopper”) which we aimed to with our analyses. Potential relationship between selected socio-economic characteristics of respondents and home-office work was analysed by performing a Chi-square test as the selected variables were all categorical. All statistical analyses were performed using SPSS [13].

## Results

The results of the study show overall considerable changes in daily activity and travel patterns due to the particularly restricted period of lockdown. Travel mode choice preferences and usage of various modes of transportation changed in favour of individual modes, in particular of cars. Furthermore, not only the usage patterns, but also attitudes toward the various modes of transportation changed. Looking into the subjective evaluation of using the transportation alternatives^3^ shows that, during the Coronavirus pandemic, people feel more comfortable using a car than any other mode of transportation. About 20% of the respondents reported to feel more comfortable being in a car compared to only 2% who stated the same for using public transport. In contrast to this, 63% stated to feel more uncomfortable by using or imagine using public transport, whereas only 5% reported the same for the car.

### Changes in shopping behaviour

**Fig. 1:**
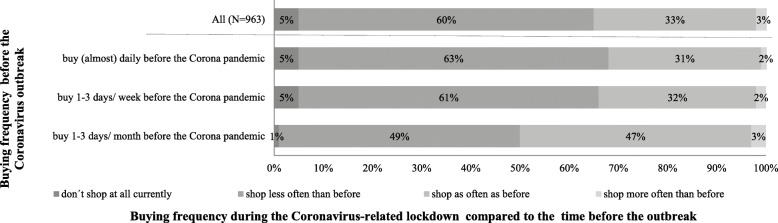
Buying frequency for daily needs in stores during the Coronavirus-related lockdown depending on buying frequency before the Coronavirus outbreak (only people who buy at least monthly in stores before the Coronavirus pandemic, *n* = 963)

Regarding shopping patterns, the results of the analyses show that, during the lockdown, about two thirds of the respondents are buying products for the daily needs (groceries, drugstore products) in stores less often than before the Coronavirus outbreak (see Fig. 1). Furthermore, the half of the people who were going for groceries before the pandemic less frequent than weekly (on 1–3 days/ month) remain going for shopping as often as before the pandemic.

**Fig. 2:**
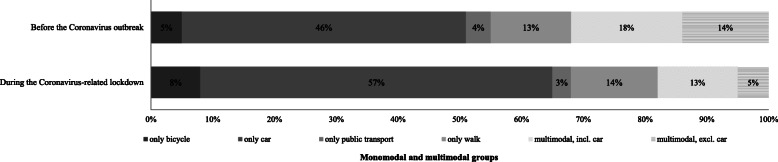
Usage patterns of different modes of transportation before the Coronavirus pandemic and during the Coronavirus-related lockdown for shopping trips (people who buy at least monthly in stores before the Coronavirus pandemic, *n* = 963)

The usage patterns of various modes of transportation during the Coronavirus-related lockdown also changed compared to the mode use before the pandemic (see Fig. 2). The majority of people (57%) used during the Coronavirus-related lockdown only the car on shopping trips; this corresponds to 11% higher share than before the pandemic. At the same time, the number of people who use also other modes of transportation, besides the car, on such trips decreased (from 18% to 13%). Furthermore, while before the Coronavirus pandemic, 14% of the people used various modes of transportation on daily shopping trips, except of the car, this number dropped to 5% during the lockdown. This suggests that during the lockdown period, people switched to monomodal use of the available modes of transportation; some of them included the car as an alternative mode of transportation for their shopping trips (although they didn’t use it before the pandemic for this trips).

**Fig. 3:**
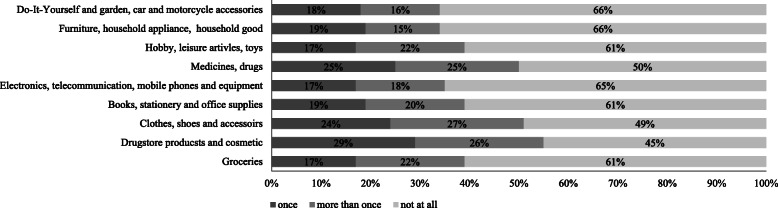
Frequency of purchase of various product types online instead in a store since the Coronavirus outbreak (only people who purchased products online because of the Coronavirus pandemic, *n* = 232)

Shopping behaviour patterns changed not only with regard to physical trips, but also in terms of online shopping behaviour. While less people bought during the lockdown period products for daily needs in stores, about one fourth of the respondents (24%) reported that because of the Coronavirus pandemic they are purchasing products, which they would usually buy in stores, online. Looking at the product types purchased online during this time period shows that the most frequently bought products were drugstore products/cosmetic (purchased by 50% of the people who shopped online during the pandemic at least once), clothes/shoes/accessories (purchased by 51% of the people at least once), and medicines (purchased by half of the subsample at least once; see Fig. 3). Furthermore, 39% of the people who bought during the lockdown products online reported that they purchased at least once groceries in online shops instead of in physical grocery stores.

As next, in order to understand better which user segments are more prone to be among the people who purchase products online instead of in stores during the lockdown, a binary logistic regression was performed. Various individual characteristics and their effects on the probability to belong to this group were analysed. The model (χ^2^(6) = 70.863, *p* < .001) and the coefficients of all selected variables, except of one, are statistically significant. The R^2^ of the model is 0.104 and *f* = 0.34 which corresponds, according to a classification proposed by Cohen [9], to a medium effect. The results of the analysis show that being female, young (under 24 years old), living in an urban area, having already experiences with online shopping (purchased at least once a year products online before the Coronavirus outbreak), and belonging to a risk groups (self-assessment) increase the probability to belong to the group of people who purchase products online during the lockdown (see Table 2). The availability of a car in the household, on the other hand, does not have a significant effect.

**Table 2:** Summary of binary logistic regression analysis for variables predicting whether an individual belongs to the group of people who purchase during the Coronavirus pandemic products online instead of in stores

Predictor	ß	SE	***P***-value	Exp(ß)
**Constant**	**−3.960**	**0.505**	0.000***	**0.019**
Gender (female)	0.483	0.157	0.002***	1.621
Age (17 to 24 years old)	1.016	0.215	0.000***	2.762
Living in urban area	0.349	0.169	0.039**	1.418
Previous experience with online shopping	2.079	0.457	0.000***	7.994
Belongs to a risk group	0.467	0.236	0.048**	1.596
Car availability in the household	0.337	0.206	0.102	1.400
−2 Log-Likelihood	1005.315 *χ*^*2*^ *= 70.863, df = 6, p < .001*			
*R*^2^	0.104			

Significant levels: *** *p* < 0.01, ** *p* < 0.05

### Changes in travel behaviour related to leisure activities and daily errands

**Fig. 4:**
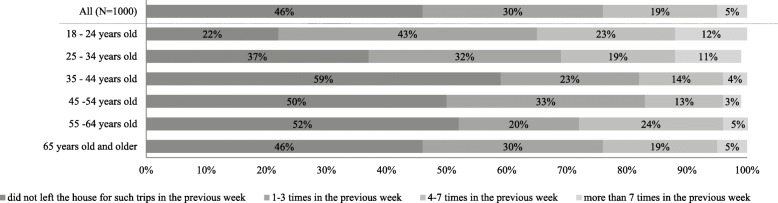
Frequency of the number of leisure trips and/ or errands in the week previous to the survey week depending on age group a respondent belongs to

Looking into further daily trips, the study data show that about half of the respondents left their house during the week before the survey for leisure and/ or to run errands (e.g. outdoor sport, doctor’s appointment, visits). There is a variation depending on age: young adults between 18 and 34 years old left more often the house than any other age group; see Fig. 4. About 78% of the people who belong to this age group reported having at least one trip for leisure and/ or to run errands. Interestingly, people in retirement age (older than 65 years) seem to leave their home in the Coronavirus-related lockdown period as frequent as people who are 35 to 64 years old.

**Fig. 5:**
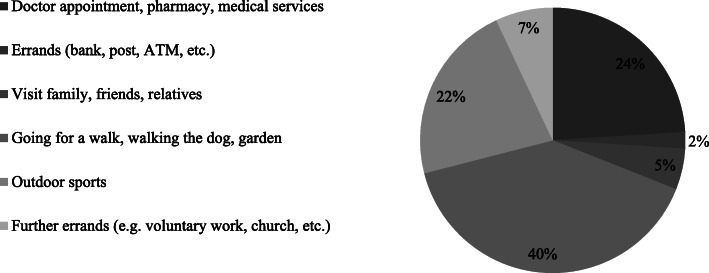
Share of people who mentioned a certain trip purpose (other than shopping or commuting) to leave home in the previous week

The most frequently mentioned purposes for leaving the house (except of shopping or commuting) was going for a walk and/ or activities in the garden (40%), followed by doctors appointment or using other medical-related services (24%) and outside sport activities (22%); see Fig. 5. Further analyses show that the trip purposes vary between different segments; the most often trips undertook in the reported week, depending on age or residential location of the respondents, were: for young adults, outdoor sport (38% of the mentioned trips); for people over 65 year, going for a walk (50% of all mentioned trips); and for people who live in urban areas, also going for a walk (43% of the mentioned trips).

**Fig. 6:**
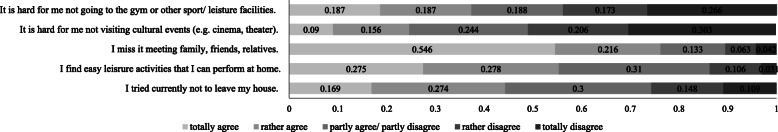
Subjective evaluation of restrictions related to leisure activities and/ or daily time-use

Besides travel behaviour patterns, also various attitudes were measured in the survey. Asked about what they miss during the lockdown time period, three fourth of the respondents reported missing meeting friends, family and relatives. This was the option with the highest agreement among the sample; see Fig. 6. Furthermore, a high share of people seems to have strategies to cope well with the contact ban and the further restrictions during the lockdown: about half of the respondents (55%) stated that they easily find leisure activities that they can perform at home. Also, 44% agreed with the statement that they are currently trying not to leave their house. Regarding typical leisure activities, such as visiting cultural events or going to the gym or to other sport/ leisure facilities, the picture of the answers on the attitudinal questions is rather heterogeneous. This could be related to less importance attached to these activities or also to different preferences for spending leisure time among the respondents.

### Changes in work-related patterns and commuting trips

**Fig. 7:**
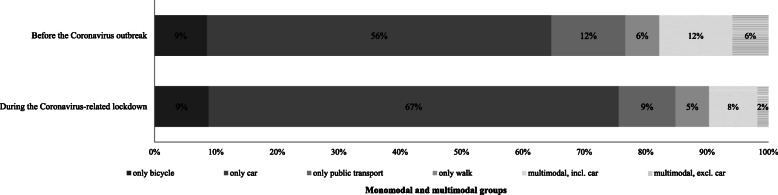
Usage patterns of different modes of transportation before the Coronavirus pandemic and during the Coronavirus-related lockdown for commuting trips (only people who had a regular commuting trip before the pandemic and still have one during the lockdown, *n* = 306)

Overall, half of the respondents belongs to the working population, i.e. they work part-time or full-time; see Table 1. The majority of the working population (69%) reported to still have a commuting trip to work. Looking at the mode use during the week reported in the survey shows a decrease in the use of public transport, partly in favour of car use; see Fig. 7. Similarly to the trends for the shopping trips during the lockdown period, the share of monomodal use of a car increased (from 56% before the Coronavirus outbreak to 67% during the Coronavirus-related lockdown), whereas the usage of various modes of transportation (multimodal use) including a car decreased. Interestingly, the share of people who use only the bicycle on their commuting trip remained the same.

As next, we looked at further changes in the work-related patterns. About 25% of the working population reported to work (at least party) in home-office during the lockdown period (10% were working only in home-office; 15% were working partly in home-office and partly at their usual working location). The results are in line with recently published statistics about the share of people in Germany who work at home during the Coronavirus-related lockdown for the same time period as considered in this study [17]. Looking at the occupation type of the people who worked in home-office during the considered period, we did not find any particular patterns or unexpected results: most of these people have an office job (e.g. sales, IT, administration); some have occupations which are usually related to the work with other people (e.g. teachers).

The most frequently mentioned reasons behind not working in home-office (also before the Coronavirus outbreak) by respondents who do not use this option were: (i) the need to have an access to machines or devices which are not available at home and (ii) working with clients or patients (both mentioned by about one third of the people who do not work in home-office).

Looking further at individual characteristics of the people who worked in a home-office shows that there is a statically significant relationship between education level and working from home (χ^2^(4) = 23.29, *p* = .000, φ = .218). While about 44% of the people with tertiary education (such as university level) were working in a home-office at least part-time, the share of people with education level lower than university who worked from home was between 15 and 19%. This might be related to the type of work performed by people with tertiary education which is mostly an office-related work. Interestingly, there was no statistically significant relationship between income level and working from home (χ^2^(7) = 7.94, *p* = .338, φ = .132) nor between amount of weekly working hours and home-office (χ^2^(2) = 1.40, *p* = .496, φ = .053).

Last but not least, the respondents who worked in home-office were asked to evaluate the work from home and to report benefits and disadvantages of it from their own perspective. Asked about the evaluation of the home-office, the majority of the people who are working during the pandemic from home (around 60%) stated to be overall satisfied with the work from home and can imagine working (more) in this way also in the future. The mentioned benefits and disadvantages were categorized in different topics – overall, nine positive and ten negative aspects of the work from home were mentioned.^4^ Among the most often mentioned benefits of home-office were (1) the flexible organization of the time when working at home and (2) the saved commute time:

*“You can organize your time somehow better and you are more motivated to work.”*

*“Very positive is that I don’t have the commuting trip with all that congestions. This saves me a lot of time.”*

*“When I finish my work at 12 o’clock, then I am immediately at home. I don’t have to plan additional commuting time anymore.”*

Also, (3) spending more time with the family and (4) having a quiet place without distraction at home were, according to the survey respondents, positive aspects of the home-office:

*“I am at home and can take care of my family. I can work more flexible.”*

*“It is wonderful. I work more concentrated and without any distractions. … It just can’t be better.”*

Further less often mentioned positive aspects were: (5) that one can do additionally his/ her household chores, (6) less stress, (7) that the virtual communication works as well as being at the office, (8) technical equipment at home is good, or (9) an overall positive evaluation of the work from home without mentioning any specific benefit.

Main disadvantages of the work from home mentioned by the respondents include (1) missing the social contact with colleagues and/or business partners as well as (2) lack of (appropriate) technical equipment at home:

*“The short exchange at the coffee machine is missing. There are mostly small things that can be done/ discussed this way.”*

*“You are then all alone and you don’t have the colleagues around you anymore. One gets lonely over the time.”*

*“Technical problems during video conferences is an issue.”*

*“Everything would be good if there were a good internet connection – not only at home, but also by my customers.”*

Moreover, some of the respondents reported that (3) the separation of work and leisure (i.e. private life) is a challenge when working from home:

*“The mix of private life and business is an issue.”*

*“I can’t switch off from the work so well.”*

Also, (4) some found it hard to concentrate only the work as they reported having more distractions at home than at the office:

*“You have to put a lot of effort not to be distracted during the work.”*

Further disadvantages mentioned by some respondents were: (5) lack of an access to relevant documents, (6) lack of work equipment at home, (7) no variety in the everyday life, (8) no clear structure of the work, (9) need to take care of the children beside the work, or (10) an overall negative evaluation without mentioning any concrete aspects.

## Discussion and conclusions

The aim of the study was to analyse changes in the travel behaviour patterns during the first Coronavirus-related lockdown in Germany, the underlying drivers of these changes besides the nationwide contact and activity restrictions and to discuss potential mid-and long-term impacts on everyday travel patterns. For this purpose, an online survey with a representative sample for Germany was conducted at the beginning of April 2020.

Generally speaking, the results of the study show that travel behaviour and mode choices changed during the Coronavirus-related lockdown period. People make fewer trips, use increasingly online shopping options, and part of the working population worked from home, i.e. in home-office. Public transport suffers from the crisis most: it is used less often than before the Coronavirus outbreak and more than a half of the respondents stated to feel uncomfortable being in a public transport during the pandemic period. Simultaneously, the use of car as a mode of transportation for daily trips increases.

Looking deeper at the activity-related travel patterns, the results of the study show firstly, that the majority of people go less often shopping for daily needs (groceries, drugstore products) than before the advent of the Coronavirus in Germany. One fourth of the people stated to purchase products (mainly drugstore products/cosmetic, clothes/ shoes/ accessories, and/ or medicines/ drugs) online instead of stores because of the Coronavirus pandemic. Several individual characteristics which make people being more prone to belong to this group were identified. People who shop online during the Coronavirus-related lockdown were rather female, young, live in urban areas, have previous experiences with online shopping and are more likely to assess their risk of Coronavirus infection as high. Secondly, about half of the adult population still left their home for leisure or to run errands (besides for shopping and/or commuting) during the strictest period of lockdown; younger people tended to leave their home for such purposes more often than older one and the purpose depends, among others, on age groups and residential location (e.g. most frequently mentioned purpose by young adult was outdoor sport, whereas the most frequently purpose for people in retirement age was going for a walk). Thirdly, the majority of the working population still had a (regular) commuting trip to work during the lockdown period; one in four of the working population works from home, i.e. in a home-office.

Several implications for current and future activity and travel pattern changes can be derived from the study results. The results suggest an increase of the importance of the car as mean of transport and at the same time decrease in the attractiveness of using public transport because of the Coronavirus outbreak. Considering environmental challenges and considering the ongoing trend toward increasing transport demand, which require the transition to a more sustainable transport system, suitable measures have to be implemented to support the use of active and collective modes of transportation or to prevent a further shift to individual motorized modes of transportation. Short-term measures which focus on decreasing the risk of infection in public transport, including the requirement to wear a mask in the vehicles and virtual information about utilization rates (for long-distance trains) have been already implemented. Further common measures discussed also in Musselwhite et al. [18] include internal cleaning and sanitation of vehicles. However, a question arises whether the additional cost for such actions can be returned with the still lower number of passengers. In other words, the economic viability of such measures has to be considered. Also, given the changed preferences towards the public transport indicated in our study, it is important to assess also if the discussed short-term actions lead to lower individually perceived infection risk by the users. On long term, additional transport policy strategies and measures that aim to facilitate the use of public transport usage might have to be developed and implemented. It is plausible to assume that the Corona crisis might increase the need for “push actions” beside pull-measures that improve the supply side of the public transport. Such additional push-measures can include, for instance, infrastructure pricing or CO_2_ taxation for car users. The effect of these and further measures for coping with the currently lower usage rates and negative perception of public transport among the users has to be, however, further evaluated. Looking at potential policy implications for active transport modes, the results of the study indicates that individual transport became increasingly important and this includes also cycling and walking. With regard to these active modes of transportation, current policy strategies or experimental projects that facilitate the use of cycling during the pandemic, such as pop-up cycling lanes, might pave the way for further development of such solutions.

Furthermore, the increasing digitalization of all areas of the daily life, the currently raising number of people who gain experience with home-office and the overall satisfaction with the work from home, shown in the study, suggest a potential for mid- and long-term changes of working activities and work-related travel. Last but not least, shopping patterns are also changing due to the Coronavirus crisis with a raise in online purchases. The mid- and long-term implications for urban shopping infrastructures are still unclear: the demand and location choice of both physical stores for various types of products and of logistic and distribution centres in urban areas might change.

The strengths of this survey are that (i) it captures changes in travel patterns during the strictest Coronavirus-related lockdown period in Germany, (ii) consider besides travel behaviour also individual strategies and attitudes related to the current situation, and (iii) due to the sampling and weighing procedure, a certain level of representativeness of the sample for the German population is ensured. A limitation, however, is that only a certain time point in the Coronavirus crisis is assessed and therefore the findings regarding travel behaviour changes and their potential mid- and long-term effects cannot be generalized for the full period of the pandemic and beyond. Moreover, the sample was recruited using a professional panel provider – an approach which suffers from some limitations that have to be also acknowledged. First, the sample consist of “part-time professional respondents”, i.e. people who receive a certain incentive to participate at the study. This might lead to a type of self-selection bias as well as underrepresentation of people in higher income classes as they might not be interested in the incentives. Furthermore, even though objective information is asked, respondents which are part of a panel might try to answer in a certain way that they think that makes it more likely to be chosen again in surveys – a bias usually more likely to occur in surveys with focus on attitudes. Besides these potential limitations, using a professional panel provider enables contacting people for further survey waves as well as using the experience of the provider as an expert for strategies to cope with these and further sampling biases. Lastly, even though the sample size is sufficient to provide a good picture of travel behaviour of the population, some of the subgroups might be too small to be representative for these target segments. For instance, the sample of people working in home-office consist of 127 of the 1000 respondents. Therefore, the results of the analyses regarding aspects of the work from home during the pandemic have to be interpreted rather as exploration of potentially relevant topics in this field that as fully representative picture of the situation of the working population in Germany.

Further research should explore the impact of the discussed changes on future behaviour patterns and also on freight transport (considering for instance the shift from physical to online shopping). As the study captures only one short phase of the Coronavirus pandemic and given the high uncertainties regarding the duration and the characteristics of the further phases of the crisis, further and longitudinal studies which track the travel behaviour changes over time are needed. This study has been designed as a longitudinal one. However, the results of the following waves are not available yet and therefore the analyses focused only on the data from the first survey wave. Future studies which follow a similar longitudinal approach can also look deeper into travel behaviour changes in urban areas, where future challenges for creating and maintaining a sustainable transport system might be bigger than in other spatial area types.

## Data Availability

The datasets generated during and/ or analysed during the current study are not publicly available due to data privacy reasons.

## Footnotes

1 This study is designed as a longitudinal study. This paper summarized the main results from the first survey wave. The second study wave has been conducted in June 2020; the third one will be conducted in 2021. Therefore, the results of the analyses of the following waves are expected to be published in 2021.

2 https://www.kantardeutschland.de

3 The survey respondents were asked the following question for various modes of transportation: *“How do you feel when using the following modes of transportation, respectively how would you feel if you had to use the following modes of transportation? Please compare your current perception with your perception before the Coronavirus spread.”.* The respondents could choose between the following five answer categories: “much more comfortable than before”, “more comfortable than before”, “no change”, “more uncomfortable than before”, “much more comfortable than before”.

4 The questions about benefits and disadvantages of working in home-office were open questions, i.e. respondents could write the answers as free text. All answers were categorized afterwards by the study team by performing a thematic analysis in Excel. Due to the small subsample size of people who reported work in home-office, we decided not reporting the share of people mentioned a certain option, but to only summarize the main results.

## References

[1] Askitas, N., Tatsiramos, K. & Verheyden, B. 2020. Lockdown strategies, mobility patterns and covid-19. arXiv preprint arXiv:2006.00531.

[2] Axhausen, K. W. (2020). *The impact of COVID19 on Swiss travel. Internet access, automation and COVID-19: On the impacts of new and persistent determinants of travel behaviour (TRAIL and TU Delft webinar 2020)*. IVT, ETH Zurich.

[3] Bitkom, E. V. (2020). *3 von 10 Verbrauchern bestellen in der Corona-Krise online Lebensmittel*.

[4] Blom, A. G., Wenz, A., Rettig, T., Reifenscheid, M., Naumann, E., Möhring, K., … Axenfeld, J. (2020). *Die Mannheimer Corona-Studie: Das Leben in Deutschland im Ausnahmezustand. Bericht zur Lafe vom 20. März bis 31. März 2020*.

[5] BMVI 2020. RegioStaR. Regionl statistical spatial typology for mobility and transport research.

[6] Budd, L., & Ison, S. (2020). Responsible transport: A post-COVID agenda for transport policy and practice. *Transportation Research Interdisciplinary Perspectives*, *6*, 100151. 10.1016/j.trip.2020.100151. 34173454 10.1016/j.trip.2020.100151 PMC7311912

[7] Cartenì, A., Di Francesco, L., & Martino, M. (2021). The role of transport accessibility within the spread of the coronavirus pandemic in Italy. *Safety Science*, *133*, 104999. 10.1016/j.ssci.2020.104999. 32952302 10.1016/j.ssci.2020.104999 PMC7489889

[8] Circella, G. (2020). The COVID-19 pandemic: What does it means for mobility? Waht are the temporary vs. longer-term impacts? In *COVID-19 pandemic: The COVID-19 pandemic: What does it mean for transportation and mobility?* Webinar: UC Davis Institute of Transport Studies, 3 Revolutions Programm.

[9] Cohen, J. (1992). Statistical power analysis. *Current Directions in Psychological Science*, *1*(3), 98–101. 10.1111/1467-8721.ep10768783.

[10] De Haas, M., Faber, R., & Hamersma, M. (2020). How COVID-19 and the Dutch ‘intelligent lockdown’change activities, work and travel behaviour: Evidence from longitudinal data in the Netherlands. *Transportation Research Interdisciplinary Perspectives*, *6*, 100150. 34171019 10.1016/j.trip.2020.100150 PMC7284275

[11] De Vos, J. (2020). The effect of COVID-19 and subsequent social distancing on travel behavior. *Transportation Research Interdisciplinary Perspectives*, *5*, 100121. 34171016 10.1016/j.trip.2020.100121 PMC7180344

[12] Eid, M., Gollwitzer, M., & Schmitt, M. (2017). *Statistik und Forschungsmethoden*.

[13] IBM Corp (2012). *Released 2012. IBM SPSS statistics for windows, version 21.0*. IBM Corp.

[14] Kraemer, M. U., Yang, C.-H., Gutierrez, B., Wu, C.-H., Klein, B., Pigott, D. M., … Hanage, W. P. (2020). The effect of human mobility and control measures on the COVID-19 epidemic in China. *Science*, *368*(6490), 493–497. 10.1126/science.abb4218. 10.1126/science.abb4218 PMC7146642 32213647

[15] Likert, R. (1932). A technique for the measurement of attitudes. *Archives of Psychology, 22,* 5-55.

[16] Mohammadian, A. K., Shabanpour, R., Shamshiripour, A., & Rahmi, E. (2020). *TRB Webinar: How much will COVID-19 affect travel behavior?* Webinar: The National Academie of Science, engineering, medicine. Transport Research Board.

[17] Möhring, K., Naumann, E., Reifenscheid, M., Blom, A. G., Wenz, A., Rettig, T., Cornesse, C. (2020). *Die Mannheimer Corona-Studie: Schwerpunktbericht zur Erwerbstätigkeit in Deutschland*.

[18] Musselwhite, C., Avineri, E., & Susilo, Y. (2020). Editorial JTH 16–the coronavirus disease COVID-19 and implications for transport and health. *Journal of Transport Health*, *16*, 100853. 10.1016/j.jth.2020.100853. 10.1016/j.jth.2020.100853 PMC7174824 32337154

[19] Nobis, C. (2014). *Multimodale Vielfalt: quantitative Analyse multimodalen Verkehrshandelns*. Humboldt Universität zu Berlin, Mathematisch-Naturwissenschaftliche Fakultät II.

[20] Schlosser, F., Hinrichs, D., Maier, B., Brockmann, D., & Rose, A. (2020). *Second report: Mobility on the rise*.

